# Vitamin D and the Immune System from the Nephrologist's Viewpoint

**DOI:** 10.1155/2014/105456

**Published:** 2014-01-22

**Authors:** Cheng-Lin Lang, Min-Hui Wang, Chih-Kang Chiang, Kuo-Cheng Lu

**Affiliations:** ^1^Department of Internal Medicine, Cardinal Tien Hospital, Yonghe Branch, New Taipei 23445, Taiwan; ^2^Division of Nephrology, Department of Internal Medicine, Cardinal Tien Hospital, School of Medicine, Fu-Jen Catholic University, 362 Chung-Cheng Road, Hsin-Tien District, New Taipei 23148, Taiwan; ^3^Division of Nephrology, Department of Internal Medicine, National Taiwan University Hospital, College of Medicine, National Taiwan University, Taipei 10002, Taiwan

## Abstract

Vitamin D and its analogues are widely used as treatments by clinical nephrologists, especially when treating chronic kidney disease (CKD) patients with secondary hyperparathyroidism. As CKD progresses, the ability to compensate for elevations in parathyroid hormone (PTH) and fibroblast growth factor-23 and for decreases in 1,25(OH)_2_D_3_ becomes inadequate, which results in hyperphosphatemia, abnormal bone disorders, and extra-skeletal calcification. In addition to its calciotropic effect on the regulation of calcium, phosphate, and parathyroid hormone, vitamin D has many other noncalciotropic effects, including controlling cell differentiation/proliferation and having immunomodulatory effects. There are several immune dysregulations that can be noted when renal function declines. Physicians need to know well both the classical and nonclassical functions of vitamin D. This review is an analysis from the nephrologist's viewpoint and focuses on the relationship between the vitamin D and the immune system, together with vitamin's clinical use to treat kidney diseases.

## 1. Introduction

Chronic kidney disease (CKD) and end-stage renal disease (ESRD) are diseases that are increasing in the 21st century. Preventing progressive deterioration in renal function and its complications remains the main challenge that nephrology needs to fulfill. CKD is defined according to the glomerular filtration rate (GFR) and/or the presence of pathological damage to the kidneys or the presence of kidney damage markers, such as proteinuria or hematuria, for 3 months [1]. Many complications are found in these patients as the GFR decline; these include fluid overload, anemia, cardiovascular disease, malnutrition, protein energy-wasting, and mineral bone disorders (MBD). In the case of MBD, hyperphosphatemia, hypercalcemia, and hyperparathyroidism contribute to the development of vascular calcification and cardiovascular disease. As CKD progresses, compensation for the elevations in parathyroid hormone (PTH) and fibroblast growth factor-23 (FGF-23) and for reduced levels of 1,25(OH)_2_D_3_ becomes inadequate, resulting in hyperphosphatemia, abnormal bone disorders, and extra-skeletal calcification. In the Kidney Disease Outcomes and Quality Initiative (KDOQI) guideline [2] and the Kidney Disease: Improving Global Outcomes (KDIGO) guideline [3], activated vitamin D or its analogues are frequently used to treat patients with secondary hyperparathyroidism and to prevent the renal osteodystrophy. Therefore, how to use vitamin D and its analogues is an important aspect of clinical nephrology.

The classical actions of vitamin D are related to mineral metabolism and skeletal health. Vitamin D regulates blood calcium, phosphate, and parathyroid hormone concentrations by actions targeting the intestines, bone, parathyroid glands, and kidneys. In addition, nonclassical roles for vitamin D, including anticell differentiation and anticell proliferative activity with respect to various cell types, have become more and more important. The anticell differentiation effect has been correlated with cancer epidemiology. Recently, serum vitamin D levels have been found to be inversely associated with many malignancies, including breast cancer [4], head and neck cancer [5], colon cancer [6], prostate cancer [7], and pancreatic cancer [8]. In a systemic review and meta-analysis, it was found that there was a moderate inverse association between 25-hydroxy vitamin D [25(OH)D] concentrations and total cancer incidence and mortality [9]. The antiproliferative properties of vitamin D have been clinically applied to the treatment of psoriasis. Using a vitamin D analogue together with steroid [10] or ultraviolet B (UVB) treatment [11] is useful when treating psoriasis.

In addition to the above, vitamin D has another important role in terms of noncalciotropic activity, its immunomodulatory effect. This immunomodulatory effect is based on the widely expressed vitamin D receptor (VDR) that is present in the immune system. This review will focus on the relationship between the vitamin D and immunity and explore current treatments using vitamin D in the clinical nephrology with the exception of mineral bone disorders.

## 2. Vitamin D Metabolism and Deficiency in Chronic Kidney Disease

Most people derive the bulk of their vitamin D from the exposure of their skin to UVB light, which is present in sunshine. The process starts with cholesterol in the skin, which is enzymatically converted to 7-dehydrocholesterol and then converted to an unstable compound, previtamin D, by the action of UVB. Nutritional sources, such as fatty fish and some types of mushrooms, also contain major forms of vitamin D, namely, cholecalciferol (vitamin D3) or ergocalciferol (vitamin D2) [12]. These are subsequently activated during a sequential 2-step process that first involves 25-hydroxylation in the liver to produce 25(OH)D and then 1-hydroxylation, which until recently was thought to occur primarily in the kidney, to produce the active product 1,25(OH)_2_D_3_ or calcitriol [13–15]. The key enzyme in this process is 1*α*-hydroxylase (CYP27B1), which is expressed primarily in proximal tubular epithelial cells of the kidney [16]. This enzyme is expressed in other parts of the kidney and in extra-renal tissues and cells as well [17]. An individual's serum 25(OH)D level is widely accepted to determine a person's vitamin D status [13, 18]. The main plasma carrier for vitamin D metabolites is vitamin D-binding protein (VBP) [19]. VBP has the highest affinity for 25(OH)D, and virtually all plasma 25(OH)D is bound to VBP [20]. The 25(OH)D-VBP complex is taken up by the proximal convoluted tubule via an endocytic receptor, megalin. The final step in the vitamin D metabolic pathway is its inactivation, a process catalyzed by 24-hydroxylase (CYP24A1) that catabolizes the conversion of both 1,25(OH)_2_D_3_ and 25(OH)D into 1,24,25(OH)_3_D and ultimately into water-soluble calcitroic acid and the inactive blood metabolite 24,25(OH)_2_D [21, 22].

In patients with CKD, serum 1,25(OH)_2_D_3_ levels decline early in the course of kidney dysfunction, even before any changes in serum calcium or phosphorus concentrations occur and prior to any rise in serum PTH levels [23, 24]. Rising FGF-23 levels may play an even greater role in controlling 1*α*-hydroxylase activity [25, 26]. Serum values of FGF-23 are regulated by circulating phosphorus levels and values increase as CKD progresses, becoming markedly elevated in individuals with end-stage kidney disease [27]. In patients with CKD, calcitriol levels are inversely related to levels of circulating FGF-23, suggesting that the hormone may play a significant role in mineral metabolism. In total, 70% to 85% patients of CKD have low levels of 25(OH)D [28–30]. As a result of the substrate-dependent process that forms 1,25(OH)_2_D_3_, a low 25(OH)D level contributes to vitamin D deficiency [31]. Many other factors may also contribute to vitamin D deficiency, including a lack of sunlight exposure, a low protein diet (lack of vitamin-D rich food), reduced 1*α*-hydroxylase activity resulting from a reduction in renal mass and tubular dysfunction [32], decreased skin synthesis of 1,25(OH)_2_D_3_ in response to sunlight compared with an individual with normal kidney function [13], loss of 25(OH)D-VBP due to heavy proteinuria [33, 34], chronic illness, diabetes [35], and various other unknown factors [36]. On the other hand, an increase in 24-hydroxylase gene expression and an increase in the clearance of 1,25(OH)_2_D_3_ with aging have also been reported [37, 38]. These findings suggest that the combined effect of a decline in the ability of the kidney to synthesize 1,25(OH)_2_D_3_ and an increase in renal metabolism of 1,25(OH)_2_D_3_ may contribute to the high prevalence of vitamin D deficiency among CKD patients.

## 3. Immune Dysregulation in CKD Patients

CKD patients and ESRD on the replacement therapy patients have significant immune dysregulation as compared with the general population and, subsequently, have a high susceptibility to infection and a high incidence of malignancy, a poorer response to vaccination, and increased levels of cardiovascular disease [39–42]. Uremia and its treatment cause immune alterations in hemodialysis patients [43]. Several factors influence the immunity of these patients, such as uremic toxin, malnutrition, chronic inflammation, vitamin D-parathyroid hormone axis alternation, and therapeutic dialysis [44–46]. Many studies have shown that both the naïve and the acquired immune systems are impaired in these patients. Due to their immunity dysregulation, these patients have more vascular calcification, accelerated atherosclerosis, a loss of appetite, increased insulin resistance, increased muscle catabolism, renal osteodystrophy, and a high prevalence of depression [47, 48]. They also have coexisting chronic immune activation (persisted hypercytokinemia and acute-phase protein response) and chronic immune suppression (a poor vaccination response and a high incidence of infection and malignancy).

Monocytes and monocyte-derived dendritic cells of CKD patients are impaired with respect to endocytosis and maturation [49], while, in parallel, uremia suppresses immune signal-induced CYP27B1 (encoding for 1*α*-hydroxylase) expression in human monocytes [50]. CKD patients have a lower percentages of peripheral CD4^+^ T lymphocytes, CD8^+^ T lymphocytes, and B lymphocytes in the blood [51]. Further, soluble B lymphocyte markers are increased in CKD patients [52], while other studies have also shown that there is an increased incidence of B cell apoptosis in these patients [53]. ESRD patients show increased apoptosis and a diminished populations of naïve and central memory T cells [54], together with impaired antigen-specific memory CD4^+^ T cells [55]. In dialysis patients, Th1 lymphocytes show decreased expression of the antiapoptotic molecule Bcl-2, which makes the Th1 cells more susceptible to apoptosis [56]. A similar decline in Th1 cell population and the enhancement in Th2 differentiation have also been noted in CKD and dialysis patients [30, 57, 58]. In addition, we have recently shown that Th17 cells are increased in chronic HD patients, whereas Treg cells are decreased (submitted). This Th17/Treg functional imbalance exists in uremic patients and is associated with the development of acute cardiovascular events including myocardial injury and microinflammation [59, 60].

Preactivated monocytes overproduce cytokines such as tumor necrosis factor-*α* (TNF-*α*), interleukin- (IL-)1, IL-6, and IL-10 [61, 62]. TNF-*α* and IL-1 are the major cytokines produced by activation of the Toll-like receptor (TLR) signaling pathway; this is the key receptor that recognizes lipopolysaccharides (LPS) [63]. In addition, IL-6, the proinflammatory cytokine, which has been shown to play a key role in atherosclerosis and protein-energy wasting, is elevated in the CKD patients [64–66]. Serum levels of IL-12 and IL-18 are both increased in CKD patients, and both of them are correlated with the inflammatory process [67, 68]. Moreover, high proinflammatory cytokine (IL-1, IL-6, and TNF-*α*) levels and low anti-inflammatory cytokine (IL-4, IL-5, and CH50) levels have also been found in hemodialysis patients [69].

**Figure 1:**
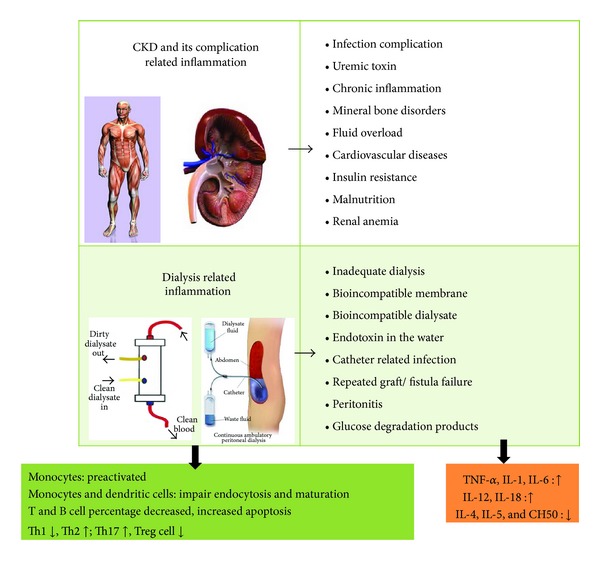
Several factors are related to immune dysregulation when renal function declines and when a patient is on renal replacement therapy.

In addition to uremic toxin, dialysis-related factors such as bioincompatibility of the hemodialysis dialyzer, the presence of endotoxins in the water, access-related infection, the presence of glucose degradation products in peritoneal dialysis solution, and the presence of advanced glycation end products are important; all of the above are able to induce chronic inflammation and will activate the immune response. Together, these findings indicate that, in general, CKD patients have immune dysregulation that includes both the cellular part and hypercytokinemia (Figure 1).

## 4. Vitamin D and the Innate Immune System

The innate immune response, which includes natural killer cells, macrophages, and their monocyte precursors, plays a central role in initial responses to pathogenic organisms and/or tissue damage. Their role is to engulf pathogens and cell debris by phagocytosis and then eliminate or assimilate the resulting waste material. The earliest evidence of vitamin D effect on innate immunity came from the treatment of tuberculosis treatment with cod liver oil, which is a major source of vitamin D [70]. The action of vitamin D on macrophages includes the ability to stimulate the differentiation of precursor monocytes into more mature phagocytic macrophages [71–73]. Macrophages have their own 1*α*-hydroxylase and require sufficient ambient levels of 25(OH)D substrate in order to generate internal 1,25(OH)_2_D_3_. Striking evidence of macrophage 1*α*-hydroxylase activity is found in granulomatous conditions such as tuberculosis, sarcoidosis, and inflammatory bowel disease, where 1,25(OH)_2_D_3 _levels may be markedly elevated [74]. In sarcoidosis patients there is increased production of 1,25(OH)_2_D_3_ despite hypercalcemia. The disordered calcium homeostasis in sarcoidosis is due to dysregulation of the production of 1,25(OH)_2_D_3_ by activated macrophages [75]. Unlike renal 1*α*-hydroxylase, the 1*α*-hydroxylase produced by macrophages is not suppressed by elevated calcium or by 1,25(OH)_2_D_3 _and is upregulated by immune stimuli such as interferon gamma (IFN-*γ*) and LPS [76, 77].

Vitamin D, vitamin D receptor, and retinoid X receptor directly activate the transcription of antimicrobial peptides such as defensin *β*2 and cathelicidin [78–80]. When monocytes are exposed to a pathogen, this will induce 1*α*-hydroxylase and the vitamin D receptor after the pathogen is recognized by the TLR, which results in production of cathelicidin [81]. This cathelicidin will cleave microbial membranes and is upregulated in response to infections in humans; it acts against bacteria, viruses, and fungi [82–84]. In some critical sepsis patients, significantly lower serum 25(OH)D and cathelicidin levels have been identified [85]. The association between a low level of cathelicidin and death from an infectious cause has also been observed in hemodialysis patients [86]. In addition, our previous study also indicated that the presence of the C allele of −1237T/C in the TLR-9 gene increases susceptibility towards development of ESRD. Thus, patients with this functional TLR-9 promoter polymorphism had a higher mean plasma IL-6 level than those carrying −1237TT [87]. In macrophages, vitamin D suppresses nuclear factor- (NF-)*κ*B activity by upregulating expression of I*κ*B through stabilization of I*κ*B-mRNA and a reduction in its phosphorylation [88, 89]. Decreased macrophage function under conditions of vitamin D deficiency has been noted in sera from patients who are vitamin-D deficient; this resulted in a lower bactericidal response compared to vitamin-D replete individuals [85]. Although vitamin D has an antimicrobial effect, it also provides feedback regulation of the immune activation pathways. 1,25(OH)_2_D_3_ has been shown to potently downregulate expression of monocytes TLR2 and TLR4, thereby suppressing inflammatory responses that are normally activated by these receptors [90].

**Figure 2:**
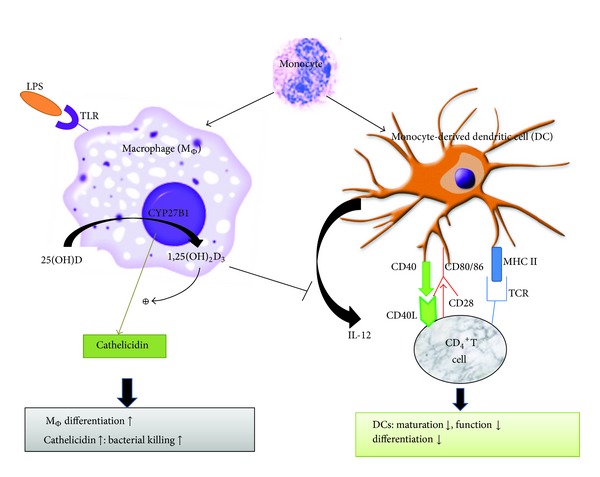
Vitamin D and innate immune system. 1,25(OH)_2_D_3_ promotes innate immunity when macrophage (M_Φ_) is activated by TLRs; CYP27B1 is induced enabling the macrophage to produce 1,25(OH)_2_D_3_, which subsequently gives rise to cathelicidin. On the other hand, 1,25(OH)_2_D_3_ inhibits the expression of costimulatory molecules (DC40, CD80/86) and major histocompatibility complex class II (MHC II) on the surface of monocyte-derived dendritic cell (DC) and inhibits the production of inflammatory cytokines, such as interleukin-12 (IL-12).

Apart from macrophages/monocytes, some other antigen presenting cells, such as dendritic cells (DCs), also express VDR and the vitamin D metabolizing enzymes, 1*α*-hydroxylase and 24-hydroxylase. Vitamin D may have an important role in promoting dendritic cell tolerogenicity via alterations in their function and morphology [91, 92]. In the presence of 1,25(OH)_2_D_3_, DCs exhibit reduced expression of major histocompatibility complex (MHC) class II molecules and various adhesion molecules (CD40, CD80, and CD86) [93–95]. This leads to reduced antigen presentation that is accompanied by a lower IL-12 secretion but an increased production of the tolerogenic IL-10; this then promotes development of Th2 lymphocyte differentiation [91]. Therefore, vitamin D inhibits the maturation and differentiation of dendritic cells; thus it might be expected that treatment with vitamin D or its analogues may reduce the immune response. Overall, 1,25(OH)_2_D_3 _is able to enhance the innate antibacterial defense capacity and create a more tolerogenic profile toward autoimmune phenomena (Figure 2).

## 5. Vitamin D and the Adaptive Immune System

Early studies demonstrated that there is expression of VDR in both T and B cells [96]. VDR expression by these cells is very low in resting conditions, but upon activation and proliferation, T cells and B cells upregulate VDR expression significantly, which influences the differentiation and proliferation of these cells [12]. Vitamin D exerts an inhibitory action on this area of the adaptive immune system.

In the T cells, 1,25(OH)_2_D_3_ plays an important role in proliferation and differentiation. Currently, four potential mechanisms by which vitamin D influences T cell function have been proposed. These are, firstly, *direct* endocrine effects via systemic 1,25(OH)_2_D_3_, secondly, *direct* intracrine conversion of 25(OH)D to 1,25(OH)_2_D_3_ by T cells itself, thirdly, *direct* paracrine effects following conversion of 25(OH)D to 1,25(OH)_2_D_3_ by local monocytes or dendritic cells, and finally, an *indirect* effect on antigen presentation to T cells which is mediated via localized APC and is affected by calcitriol [97]. Vitamin D promotes a T cell shift from Th1 to Th2, which might help to limit potential tissue damage associated with Th1 cellular immune responses. Treatment of T cells with calcitriol or analogues inhibits the secretion of the proinflammatory Th1 (IL-2, IFN-*γ*, and TNF-*α*), Th9 (IL-9), and Th22 (IL-22) cytokines [98–101] but promotes the production of more anti-inflammatory Th2 cytokines (IL-3, IL-4, IL-5, and IL-10) [30, 102, 103]. Active vitamin D can modulate Th2-cell responses both indirectly, through suppression of IFN-*γ* and IL-2 in Th1 cells, and directly by influencing expression of Th2 cytokines such as IL-4.

**Figure 3:**
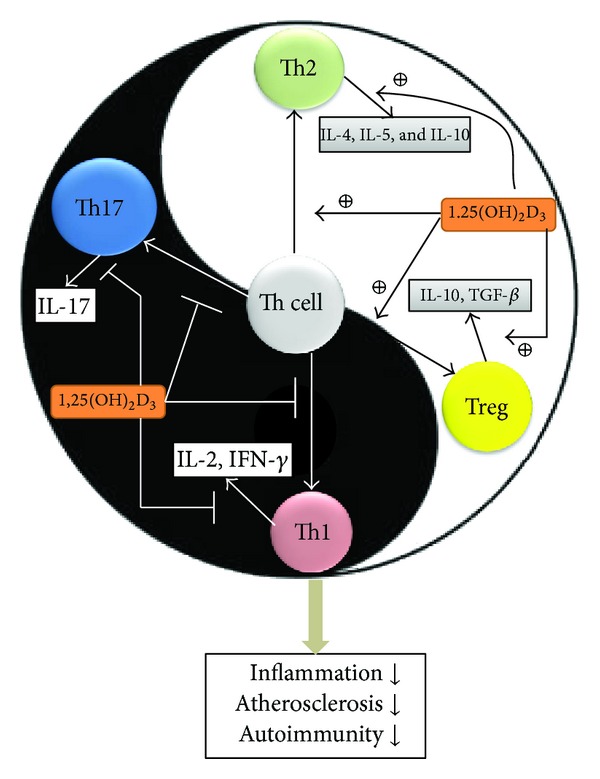
Vitamin D and adaptive immune system. The adaptive immune system is like Tai-Chi, namely, that it separates the Yin and the Yang. 1,25(OH)_2_D_3_ directly modulates T cell responses and polarization related to the inflammatory molecules Th1 and Th17 in order to give rise to protective Th2 and Treg cells. In addition, 1,25(OH)_2_D_3_ also inhibits the inflammatory Th1 and Th17 cytokines and upregulates the protective Th2 and Treg cytokines. When these effects are integrated, the adaptive immune system may produce lower levels of inflammation, atherosclerosis, and autoimmunity.

1,25(OH)_2_D_3_ reduces expression of IL-17 [104]. IL-17-producing Th17 cells play a crucial role in the induction of autoimmune disease and inflammation [105]. T cell exposed to 1,25(OH)_2_D_3 _produced significantly decreased levels of IL-17, IFN-*γ*, and IL-21 and has significantly increased expression of genes typical for regulatory T cells (Tregs) [3]. The Treg cells have an anti-inflammatory role and control autoimmune diseases by releasing IL-10 and TGF-*β* [106]; in addition, Treg cells are able to be induced and stimulated by 1,25(OH)_2_D_3_ though an indirect pathway, via APCs and DCs, or through a direct pathway, via an endocrine effect or the intracrine conversion of 25(OH)D to 1,25(OH)_2_D_3_ by Treg cells themselves [107–109]. Thus, 1,25(OH)_2_D_3_ exerts a broad range of effects on inflammation and autoimmune disease by reducing Th17 cell numbers and by having effects that are beneficial in terms of autoimmune and host-graft rejection; these events occur by enhancing Treg cell numbers. However, the regulation of T cells may come at a price because it leads to a decreased response to pathogens and to a reduction in immune surveillance. 1,25(OH)_2_D_3_ is able to significantly alter the behavior of the T cells, favoring the development of tolerance via an increase in Th2 and Treg cell activity and a reduction in proinflammatory Th1 and Th17 cell activity (Figure 3).

VDR is also expressed in inactivated B cells [110]. In B cells, 1,25(OH)_2_D_3_ plays an antiproliferative role involving an inhibition of cell differentiation, an inhibition of cell proliferation, reduced initiation of apoptosis, and decreased immunoglobulin production. These effects are probably indirectly mediated by T cells [111, 112]. This control of B cell activation and proliferation is important in autoimmune diseases due to the fact that B cells producing autoantibodies that play a major role in the pathophysiology of autoimmune disease, such as systemic lupus nephritis, type 1 diabetes, inflammatory bowel disease, and multiple sclerosis.

## 6. Vitamin D Alters Immunity in Clinical Nephrology Patients

The calciotropic action of vitamin D is its major use in clinical nephrology. As CKD progresses, compensation for the elevations of PTH and FGF-23 as well as the decreased levels of 1,25(OH)_2_D_3_ becomes inadequate, resulting in hyperphosphatemia, hypocalcemia, abnormal bone disorders, and extra-skeletal calcification. Recent studies have unraveled some of the complications that are present in ESRD patients, including anemia [113, 114], lipid and insulin abnormalities, cardiovascular risk [115, 116], and overall mortality [117–119]; these are able to be improved by correcting for the patient's vitamin D deficiency. 1,25(OH)_2_D_3_ has been proven to have an antiproteinuric effect and to interfere with the renin-angiotensin-aldosterone system (RAAS). 1,25(OH)_2_D_3_ is able to maintain the structural and functional integrity of podocytes [120–123] and also suppresses directly renin expression at the transcriptional level [124–126]. Studies have shown that a combination of vitamin D or its analogue with RAAS blockade agents is able to ameliorate renal fibrosis [127]. The renoprotective effects of vitamin D and its analogues include suppression of the RAAS and a reduction in proteinuria; these may occur either directly through the protection of podocytes or via negative regulation of the RAAS. The anti-inflammatory properties of 1,25(OH)_2_D_3_ may be attributed to a suppression of the NF-*κ*B pathway. The NF-*κ*B pathway plays an important role in the progression of renal disease because it promotes both inflammation and fibrogenesis via regulation of various inflammatory cytokines (MCP-1, TNF-*α*, and PAI-1) [128].

**Figure 4:**
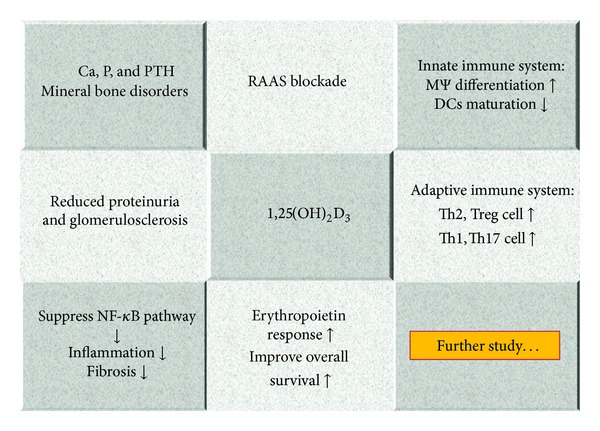
Overview of biological functions of vitamin D in clinical nephrology.

Many studies have focused on treatment with 1,25(OH)_2_D_3_ to alter immune function in CKD and ESRD patients. 1,25(OH)_2_D_3_, when used in HD patients with secondary hyperparathyroidism, is able to enhance Th2 cell differentiation [30] and decrease IL-6 expression [129]. It also can attenuate inflammatory and oxidative stress in HD patients [130]. Among dialysis patients, a low serum level of 25(OH)D is correlated with a high panel of reactive T cell values, which means that vitamin D deficiency is related to a poor posttransplant outcome [131]. When there is acute kidney injury, vitamin D deficiency seems to predispose individuals towards an increased risk of sepsis, endothelial dysfunction and also prevents the healing of renal ischemia-reperfusion injury via the TLR, NF-*κ*B, and the RAAS pathway [132]. In HD patients, treatment with vitamin D and its analogues is able to reduce platelet activating factor/thrombin activity and metabolism as well as lower serum IL-6, IL-8, IL-1*β*, and TNF-*α* levels, all of which are inflammatory markers [133, 134]. In terms of its noncalciotropic effects, 1,25(OH)_2_D_3_ is able to significantly alter the behavior of T cells; this favors the development of tolerance and a reduction in proinflammatory activity, while at the same time ameliorating renal fibrosis and slowing down the development of proteinuria (Figure 4).

## 7. Conclusions

As nephrologists, we are continually looking for ways to improve the immune system of patients and patient outcome. The broad tissue distribution of 1*α*-hydroxylase and vitamin D receptor has established a role for 1,25(OH)_2_D_3_ in the pathophysiology of many diseases and this has provided a therapeutic role for the 1,25(OH)_2_D_3_. Growing evidence indicates that the usefulness of vitamin D extends beyond its classical role in maintenance of mineral homeostasis and, in this context, the present use of active vitamin D includes the treatment of secondary hyperparathyroidism in CKD. Moreover, vitamin D deficiency is common among CKD patients and in fact may contribute to deterioration in their kidney function. In addition to the traditional supplementation of CKD patients with 1,25(OH)_2_D_3_, it is possible that, by assessing and reducing any 25(OH)D deficiency and treating secondary hyperparathyroidism, physicians may be able to adequately fuel both the renal and extra-renal pathways of 1,25(OH)_2_D_3_ synthesis. This will maintain both the classical and nonclassical functions of vitamin D and ultimately influence the clinical outcomes of this high-risk group of patients.
